# Effectiveness of a dietary intervention on macronutrient intake, lean mass and strength gains in males participating in a supervised resistance training program

**DOI:** 10.1186/1550-2783-8-S1-P20

**Published:** 2011-11-07

**Authors:** Jonathan Oliver, Michelle Mardock, Andrew Jagim, Adam Sanchez, Julie Kresta, Stephen Crouse, Richard Kreider

**Affiliations:** 1 Exercise & Sport Nutrition Lab, Texas A&M University, College Station, TX 77843, USA

## Background

ISSN recommendations for individuals involved in a general fitness program are to ingest 25-35 kcal/kg/day consisting of 3-5 g/kg of carbohydrate and ≤30% of total calories from fat. Additionally, the ISSN recommends that individuals engaged in resistance-training should ingest 1.4-2.0 g/kg/d of protein and to ingest some protein after exercise. This study examined whether nutritional counseling and post-workout supplementation affects dietary intake during training.

## Methods

Eleven trained men (25±5 yrs, 180±6 cm, 82±12 kg, 14±3 %fat, training 7±4 years, 3±2 days/wk) were provided nutritional counseling by a dietitian prior to participating in a supervised resistance-training program (4 days/wk). A supplement containing 40g carbohydrate, 20g protein, and 3.5g fat was provided post-exercise. Diet records were obtained at 0, 3, 7, & 11 weeks while DEXA determined body composition, 1RM bench press, and 1RM squat measurements were obtained at 0, 4, 8, & 12 wks. Data were analyzed by ANOVA with repeated measures and are presented as means ± standard deviations.

## Results

Nutritional counseling did not change energy intake (30.9±5.5, 36.4±9.6, 35.0±10.2, 33.1±6.1 kcal/kg/day; p=0.20) or fat intake (34±10, 34±6, 34±6, 34±7 %; p=0.97). Protein intake significantly increased from baseline (1.7±0.4, 2.4±0.8, 2.3±0.6, 2.4±0.5 g/kg; p=0.002) while carbohydrate intake significantly decreased (3.5±1.2, 3.3±0.6, 2.8±1.2, 2.3±0.9 g/kg; p=0.02); corresponding to an increase in percentage of protein (22±6, 26±3, 28±10, 29±6 %; p=0.03) and a decrease in percentage of carbohydrates (45±15, 38±8, 31±10, 28±9 %; p=0.003). After 4, 8 and 12 weeks, respectively, a significant increase in lean mass was observed (1.3±1.7, 2.1±1.8, 2.2±2.1 kg; p=0.001) with no significant effect on body fat percentage (14.3±2.7, 15.0±3.3, 14.7±3.5, 15.1±3.5 %; p=0.34). Bench press 1RM (-2±6, 3±6, 9±5 %; p=0.001) and squat 1RM (14±10, 33±14, 43±18 %; p=0.001) increased from baseline.

## Conclusion

Nutritional counseling prior to engaging in a resistance-training program that included post exercise supplementation increased dietary protein intake and resulted in positive training adaptations despite a reduction in carbohydrate intake. Additional nutritional guidance may be necessary to ensure adequate carbohydrate intake particularly in athletes engaged in heavy training.

## Funding

Supported by National Strength and Conditioning Association. Supplements provided by Cytosport^TM^, Inc.

